# Age- and Cell-Specific Regulation of Testicular Polyamine Metabolism Promotes Increased Catabolism During Maturation and Aging in Syrian Hamsters

**DOI:** 10.3390/biology15141184

**Published:** 2026-07-17

**Authors:** Alina Cavallotti Gomez, Imanol González, Soledad Paola Rossi, Ricardo Saúl Calandra, Mónica Beatriz Frungieri, María Eugenia Matzkin

**Affiliations:** Instituto de Biología y Medicina Experimental, IBYME-CONICET, Ciudad de Buenos Aires 1428, Argentina

**Keywords:** testis, testicular cell populations, putrescine, spermidine, spermine, polyamine-monoacetylated derivatives

## Abstract

No previous studies have characterized testicular polyamine metabolism across specific cell populations, including Sertoli cells, testicular peritubular cells, Leydig cells, germ cells, and testicular macrophages, during testicular maturation and aging. Here, we used immature, young adult, and aged adult Syrian hamsters as an animal model to quantify the main polyamines (PA) and their monoacetylated derivatives at the tissue and cellular levels by thin-layer chromatography and to assess the expression profiles of key genes involved in polyamine metabolism by RT-qPCR. At the tissue level, total PA concentration increased with age. During testicular maturation, putrescine increased and spermine decreased, whereas aged testes showed spermidine predominance. Testicular cell populations contributed unequally to PA production and release, and these contributions changed markedly with age. At the cellular level, aging was associated with a shift toward spermine- and N^1^-spermine-rich profiles, suggesting enhanced PA catabolism, likely supported by Sat1/Paox up-regulation. Thus, PA catabolic enzymes represent potential therapeutic targets for aging-associated or even idiopathic infertility-related testicular alterations.

## Simple Summary

Polyamines are small polycationic molecules that can control multiple cellular processes, yet they can become toxic when their biosynthesis and/or catabolism is deregulated. No previous studies have examined the complex metabolism of polyamines in the testis during development and aging while discriminating among its different cell populations. Using the Syrian hamster as our experimental model, we found that different cell populations (Sertoli cells, testicular peritubular cells, Leydig cells, germ cells, and testicular macrophages) contribute unequally to testicular polyamine production and release. These contributions changed substantially with age in immature, young adult, and aged adult hamsters. The age-related remodeling of intracellular and extracellular polyamine content showed more dissimilar abundance patterns in immature animals and less dissimilar abundance patterns in adult aged animals. Characterizing the predominant and/or deficient polyamines in each testicular cell type across developmental ages will support future studies on their general and cell-specific roles in testicular physiology and could potentially reveal new therapeutic candidates to slow testicular aging or even alleviate infertility-related testicular alterations.

## 1. Introduction

Polyamines (PAs) are non-protein nitrogenous compounds of low molecular weight, present in both eukaryotic and prokaryotic cells. Structurally, PAs are polycationic molecules composed of several amino groups along hydrocarbon chains. They can be classified as aliphatic, aromatic, or heterocyclic amines. The most abundant are aliphatic amines, among which putrescine, spermidine, and spermine are their major representatives in mammals, reaching very high intracellular concentrations, ranging from micromolar to low millimolar [1,2,3,4,5]. They interact with nucleic acids, proteins, and lipids, thus influencing key cellular processes like gene expression, cell growth, proliferation, cell differentiation, response to stress, and redox balance [5]. Despite their various physiological functions and cellular needs, PAs can become toxic to cells, either due to their high concentrations or via their catabolism, which leads to the production of highly toxic intermediaries such as aldehydes, peroxides, and ammonia [4,5]. This is why PA metabolism has evolved to exhibit multiple feedback regulatory mechanisms to secure a highly robust metabolic module that ensures the maintenance of PA levels within the limits compatible with cellular homeostasis in different circumstances [1,2,3,4,5].

**Figure 1:**
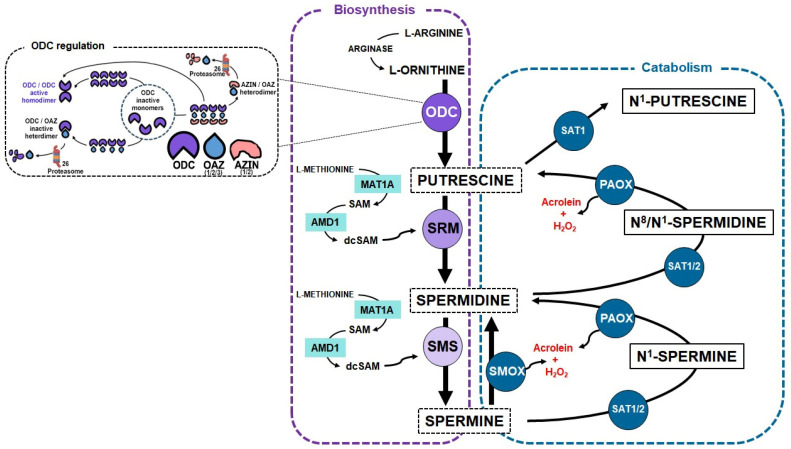
Schematic representation of polyamine metabolism. ODC: ornithine decarboxylase; SRM: spermidine synthase; SMS: spermine synthase; SAT1: spermidine/spermine N^1^-acetyltransferase 1; SAT2: spermidine/spermine N^1^-acetyltransferase 2; SMOX: spermine oxidase; PAOX: polyamine oxidase; MAT1A: methionine adenosyltransferase 1a; AMD1: S-adenosylmethionine decarboxylase; OAZ1: ornithine decarboxylase antizyme 1; OAZ2: ornithine decarboxylase antizyme 2; OAZ3: ornithine decarboxylase antizyme 3; AZIN1: antizyme inhibitor 1; AZIN2: antizyme inhibitor 2; SAM: S-adenosylmethionine; dcSAM: decarboxylated S-adenosylmethionine. ODC is active as a homodimer and exists in a rapid state of association/disassociation. Antizymes (OAZ1/2/3) are ODC analogs that lack enzymatic activity, while antizyme inhibitors (AZIN1/2) are also homologous with ODC but their affinity for OAZ is higher than for ODC. High concentrations of PAs lead to an increased abundance of OAZ, which benefits the formation of ODC/OAZ heterodimers, which inhibit ODC function because they are degraded by the 26S proteasome in a ubiquitin-independent mechanism. When both OAZ and AZIN are present, OAZ has a greater affinity for AZIN than for ODC, allowing functional ODC dimers to form [1,2,3,4,5,9]. The figure was created using Adobe Photoshop (Version 2026).

Although PAs can be obtained through diet and other alternative sources, such as the gut microbiota [6,7,8], all eukaryotic cells are capable of synthesizing the three major PAs [1,2,3]. In eukaryotic cells, the biosynthesis of PAs requires precursors such as L-ornithine and S-adenosylmethionine (SAM). L-ornithine is decarboxylated by the enzyme ornithine decarboxylase (ODC) to synthesize putrescine, a diamine and a precursor of all PAs (Figure 1). The synthesis of spermidine and spermine requires the action of two specific enzymes: spermidine synthase (SRM) and spermine synthase (SMS), respectively. Each of these enzymes catalyzes the sequential addition of aminopropyl groups to putrescine or spermidine. The aminopropyl group donor is decarboxylated SAM, which is formed from L-methionine by the combined action of methionine adenosyltransferase (MAT1A) and S-adenosylmethionine decarboxylase (AMD1) [1,2,3,4,5]. Spermidine and spermine levels are also regulated by the presence of a distinctive back-conversion pathway via spermidine/spermine N^1^-acetyltransferase (SAT1/SAT2) and polyamine oxidase (PAOX). The SAT1/SAT2-mediated acetylation of PAs is a rate-limiting step in their catabolism, and the expression and activity of SAT1/SAT2 are regulated at multiple levels. The oxidation of spermine by spermine oxidase (SMOX), a highly inducible enzyme, is another major mode of PA catabolism, through which spermine can be directly converted back to spermidine [1,2,3,4,5]. In addition to the many enzymes involved in PA synthesis/catabolism/interconversion, ODC is further tightly regulated. Its active form is a homodimer. However, an intricate balance of antizymes (OAZ1/2/3) and antizyme inhibitors (AZIN1/2) is involved in the maintenance of active ODC homodimers [4,5,9] (Figure 1).

There is evidence indicating that Odc is expressed in Leydig cells, Sertoli cells, and germ cells in immature rats and mice [10,11,12,13,14]. A circadian rhythm of Odc activity in the rat testis was also described [15]. The generation of transgenic mice that overexpressed ODC in the testis revealed intense morphological and functional alterations in this gonad, including fewer Leydig cells, arrested spermatogenesis, and loss of germ epithelium to various degrees—even being so drastic as to resemble a syndrome causing infertility in men known as Sertoli cell-only syndrome [16,17]. Additional studies using this mouse model led to the suggestion that putrescine may have effects on DNA synthesis during spermatogenesis and that an excessive amount of this diamine could be related to a decrease in fertility [18]. Odc expression was detected in proliferating Sertoli cells, as well as in Leydig cells, while AZIN2 expression was only detected in Leydig cells in immature rats [19,20]. Further studies in human testes revealed that AZIN2 could be detected in expressed in Leydig cells but not in the germinal epithelium [20]. Odc inhibition reduced testosterone production in rodent testes, thus suggesting a requirement for PAs for steroidogenesis [20], while exogenous spermine induced lactate production in Sertoli cells of immature Syrian hamsters [21]. Regarding the characterization of polyamine metabolism in the testis, most of these older studies have focused on analyzing the effects of reproductive hormones (e.g., FSH, androgens, and prolactin) on the expression and/or activity of ODC enzyme [10,11,12,14,21,22,23,24]. These studies were performed in rats and mice no older than 90 days and mainly focused on only three populations of the testis: Leydig cells, Sertoli cells, and germ cells. To this day, other testicular cell populations (e.g., testicular peritubular cells and macrophages) are completely neglected. Moreover, culture conditions greatly influence PA homeostasis. For example, it is now known that fetal bovine serum (FBS) contains amine oxidases that can further oxidize spermine and that this enzymatic activity is not observed in serum from humans or non-ruminants [4]. Therefore, most (if not all) of the results obtained for testicular fragments and primary cell cultures of Leydig cells, Sertoli cells, or germ cells previously mentioned need to, at least, be reviewed.

Advanced research in genomic–transcriptomic alignment, comparing the transcriptomes from 15 tissues of the Syrian hamster, has revealed that these rodents share patterns of alternative splicing modes and certain stress or metabolic responses that are more similar to those in humans compared to rats and mice [25]. Our group has previously reported that aged (22-month-old) Syrian hamsters’ testes show clear evidence of pro-inflammatory (increased NLRP3, IL1β, COX2, PGD2, testicular macrophage cell number) and pro-oxidative status (increased lipid peroxidation, CAT expression) that lead to decreased autophagy (increased p62 and decreased LAMP2) and DNA repair capability (reduced γH2AX) [26,27,28]. Very similar alterations in testicular physiology/morphology to those reported in aged Syrian hamsters have also been described by our group in human testicular biopsies from men suffering from idiopathic infertility (primarily Sertoli cell-only syndrome and hypospermatogenesis) [29,30,31,32,33,34,35].

Thus, with no previous studies focusing on understanding and deepening the knowledge of the complex metabolism of PAs in the testis while discriminating between the different cell populations, the aims of this study were (1) to analyze the testicular levels of the main PAs and their monoacetylated derivatives (AC-PAs) in immature, young adult, and aged adult Syrian hamsters; (2) to identify the testicular cell populations that contribute (and to what extent) to the local production of PAs and AC-PAs in immature, young adult, and aged adult Syrian hamsters (using primary cell cultures of Sertoli cells, testicular peritubular cells, Leydig cells, germ cells, and testicular macrophages); and (3) to analyze the expression profile of the key genes involved in PA synthesis/conversion/catabolism in testicular homogenates and isolated cell populations from the immature, young adult, and aged adult Syrian hamster.

## 2. Materials and Methods

### 2.1. Animals

Male Syrian hamsters (*Mesocricetus auratus*) were raised in our Animal Care Facility (Charles River descendants, Animal Care Lab., IBYME-CONICET, Buenos Aires, Argentina) and kept from birth to adulthood in rooms at 23 ± 2 °C under a long-day (LD) photoperiod (14 h light, 10 h darkness; lights on 07:00–21:00 h). Animals had free access to water and Purina formula chow. Male hamsters aged 21 days (immature), 90 days (young adults), and 22 months (aged adults) were killed by asphyxia with carbon dioxide (CO_2_). At the time of sacrifice, the testes were dissected and either used for the purification of different testicular cell populations (see Section 2.2, Section 2.3, Section 2.4 and Section 2.5) or stored immediately at −80 °C for PA and AC-PA quantification by thin-layer chromatography (TLC) assays (see Section 2.7) or RNA extraction followed by real-time quantitative reverse transcription polymerase chain reaction (RT-qPCR) analyses (see Section 2.8). All animal procedures were performed according to the regulations for the use of laboratory animals of the National Institute of Health (NIH, Bethesda, MA, USA) and approved by the Institutional Animal Care and Use Committee of IBYME-CONICET (CICUAL Protocol N° CE-07/2023; Approved 18 April 2023; PI: Dr. Matzkin ME).

### 2.2. Purification of Leydig Cells and Germ Cells from Immature, Young Adult, and Aged Adult Syrian Hamster Testes

Both cell populations were purified using a modified protocol previously described by our group [36,37]. Pools of 6 testes (immature animals) or 2 testes (young and aged adult animals) were assayed. Briefly, testicular cell suspensions (obtained by 2 min, 2.5 mg/mL trypsin [1300 BAEE units/mg] digestion at 34 °C followed by subsequent filtration using a 100 μm Nylon cell strainer) were subjected to a discontinuous (20–35–40–60–90%) Percoll density gradient. Leydig cells (LC) were recovered from the 40–60% interphase, while germ cells (GC) were collected from the 35–40% interphase. Cell viability was higher than 90%. Cells were washed twice in Hank’s Balanced Salt Solution (HBSS) and used for in vitro incubations (see Section 2.6) followed by TLC assays (see Section 2.6 and Section 2.7), RNA extraction followed by RT-qPCR analyses (see Section 2.8), or immunocytochemistry analyses (see Section 2.9).

### 2.3. Purification of Testicular Macrophages from Immature, Young Adult, and Aged Adult Syrian Hamster Testes

Testicular macrophages (tMAC) were purified as previously described by our group [27]. Pools of 6 testes (immature animals) or 2 testes (young and aged adult animals) were assayed. Briefly, testicular cell suspensions (obtained by 2 min, 0.2 mg/mL [125 CDU/mg] collagenase type I digestion, at 34 °C) were seeded into 35 mm culture dishes and allowed to differentially attach for 2 h (in 10% FBS, RPMI1640 at 37 °C; 5% CO_2_–95% air). Unattached cells were discarded by gentle washing, while attached cells were enriched with tMAC with cell viability higher than 90%. Cells were washed twice in HBSS solution and used for in vitro incubations (see Section 2.6) followed by TLC assays (see Section 2.6 and Section 2.7), RNA extraction followed by RT-qPCR analyses (see Section 2.8), or immunocytochemistry analyses (see Section 2.9).

### 2.4. Purification of Testicular Peritubular Cells and Sertoli Cells from Immature Syrian Hamster Testes

Both cell populations were isolated using the protocol previously validated by our group [37,38]. Pools of 6 testes were employed. Testicular peritubular cells (TPC) were purified by two consecutive short enzymatic digestions (2 min, 2.5 mg/mL trypsin and 5 min 0.2 mg/mL collagenase type I, at 34 °C) and multiple decantation steps. For Sertoli cell (SC) purification, a third enzymatic digestion step (8 min, 0.2 mg/mL collagenase type I, at 34 °C) followed by an additional 30 min decantation step was required. In both cases, testicular cell suspensions were seeded into 35 mm culture dishes and allowed to attach for 48 h at 37 °C; 5% CO_2_–95% air in RPMI1640 (Gibco, Waltham, MA, USA) supplemented with 10% FBS (Natocor, Córdoba, Argentina). Unattached cells were discarded by gentle washing, while attached cells were enriched with either TPC or SC, respectively, with cell viability higher than 90%. Cells were washed twice in HBSS solution and used for in vitro incubations (see Section 2.6) followed by TLC assays (see Section 2.6 and Section 2.7), RNA extraction followed by RT-qPCR analyses (see Section 2.8), or immunocytochemistry analyses (see Section 2.9).

### 2.5. Purification of Testicular Peritubular Cells and Sertoli Cells from Young Adult and Aged Adult Syrian Hamster Testes

Both cell populations were purified using a modified protocol that combined multiple enzymatic digestions and a discontinuous Percoll density gradient. The modified protocol was based on previously reported methodology described in adult rats and mice by other authors [39,40,41]. In the adapted protocol, pools of 2 testes were subjected to 2 min, 2.5 mg/mL trypsin digestion (at 34 °C) followed by multiple decantation steps in HBSS solution. The decanted seminiferous tubules were subjected to a second enzymatic digestion step (5 min, 0.2 mg/mL collagenase type I, at 34 °C) and a further 10 min decantation step. From here on, the protocols differed for adult TPC and SC purification.

For TPC purification, the resulting shortened seminiferous tubules that were decanted were subjected to a further 30 min decantation step followed by a third short enzymatic digestion step (5 min, 0.2 mg/mL collagenase type I, at 34 °C) and an additional 10 min decantation step. Supernatants were filtered through a 100 μm nylon cell strainer and subsequently subjected to a discontinuous (20–25–40–60–90%) Percoll density gradient. TPC were recovered from the 25–40% interphase since it represented the fraction with the highest purity level and the lowest contamination level with other cell populations (25%, 40%, and 25–40% interphase fractions were analyzed by immunocytochemistry). Cell viability was higher than 90%.

For SC purification, the resulting shortened seminiferous tubules that were decanted were subjected to a third long enzymatic digestion step (1 h, 0.2 mg/mL collagenase type I, at 34 °C, in an oscillating shaker) until the single-cell suspension stage was achieved. Three additional decantation steps were performed (3 min centrifugation at 600× *g*). The final decantation pellet was filtered through a 100 μm nylon cell strainer and subsequently subjected to a discontinuous (20–35–40–60–90%) Percoll density gradient. SC were recovered from the 35–40% interphase since it represented the fraction with the highest purity level and lowest contamination level with other cell populations (35%, 40%, and 35–40% interphase fractions were analyzed by immunocytochemistry). Cell viability was higher than 80%.

In both cases, cells were washed twice in HBSS solution and used for in vitro incubations (see Section 2.6) followed by TLC assays (see Section 2.6 and Section 2.7), RNA extraction followed by RT-qPCR analyses (see Section 2.8), or immunocytochemistry analyses (see Section 2.9).

### 2.6. In Vitro Incubations

In vitro incubations of testicular cell populations (SC, TPC, LC, GC, and tMAC) obtained from immature, young adult, or aged adult Syrian hamsters were carried out at 37 °C (5% CO_2_—95% air) for 1 h in basal conditions (RPMI1640 medium, fetal bovine serum-free). Cells were used either for TLC assays (see Section 2.7) or RNA extraction followed by qRT-PCR (see Section 2.8). Media were collected and used for TLC assays (see Section 2.7).

### 2.7. Polyamines and Monoacetylated Derivatives Thin-Layer Chromatography Assays

Polyamines (PAs) and monoacetylated derivatives (AC-PAs) levels were assayed by thin-layer chromatography (TLC) according to the protocol previously validated in our laboratory [42,43], with some modifications. TLC was used to analyze the testicular tissue levels as well as intracellular and extracellular levels of PAs and AC-PAs in different testicular cell populations from immature, young adult, and aged adult Syrian hamsters. Testes (approximately 100 mg) and cell samples (approximately 1–3 × 10^6^ cells or the corresponding culture media volume) were depleted of protein content (by protein precipitation with 0.4 N perchloric acid in a 1:1 ratio). The resulting supernatants (as well as freshly prepared PAs and AC-PAs standards) were subjected to the formation of fluorescent polyamine–dansyl chloride derivatization products in a reaction performed at room temperature overnight with 8 mg/mL dansyl chloride in a Na_2_CO_3_-saturated solution. TLC separation of dansylated standards and samples was performed in 20 × 20 cm aluminum silica gel plates (Merck Millipore #5553, Burlington, MA, USA). Standard curves for putrescine, spermidine, spermine, N^1^-acetylputrescine, N^8^-acetylspermidine, N^1^-acetylspermidine, and N^1^-acetylspermine (N^1^-Pu, N^8^-Sd, N^1^-Sd, N^1^-Sp, respectively) were run simultaneously (7.8125–15.625–31.25–62.5–125–250–500 × 10^−4^ nmol/spot). In every case, plate development was carried out in darkness, at room temperature, in a glass tank containing 100 mL of the corresponding mobile phase. For putrescine, spermidine, and spermine resolution, a single run in chloroform/triethylamine (9:1) was performed. For N^1^-Pu, N^8^-Sd, N^1^-Sd, and N^1^-Sp separation, two consecutive runs in 100% ethyl acetate, followed by a run in chloroform/methanol (95:5), were performed. Spot positions for standards and samples were photographed under UV light, followed by densitometry. Testicular levels (PAs/AC-PAs) were expressed as millimolar (mM), while intracellular/extracellular levels (PAs/AC-PAs) were expressed as pmol/10^6^ cells. For testicular PAs/AC-PAs concentrations, the initial nmol/g tissue levels were converted to mM considering the mean testicular density for each age group: 1.08 g/cm^3^ (immature) and 1.05 g/cm^3^ (young adult and aged adult). The testicular density was calculated by dividing the testicular weight by the testicular volume. At the time of sacrifice, the testes were dissected, freed from adherent tissues, and weighed on an analytical scale (Mettler Instrument AG, Schwerzenbach, Switzerland). The testicular volume of each removed testis was estimated directly by water displacement (Archimedes principle) [44]. The mean testicular weight for each age group was 0.17 g (immature), 1.78 g (young adult), and 1.50 g (aged adult). The mean testicular volume for each age group was 0.16 cm^3^ (immature), 1.70 cm^3^ (young adult), and 1.43 cm^3^ (aged adult).

### 2.8. RT-qPCR Analyses

Total RNA was extracted from testicular fragments or purified testicular cell populations (SC, TPC, LC, GC, and tMAC) obtained from immature, young adult, or aged adult Syrian hamsters using Prepzol Reagent (Inbiohighway, CABA, Argentina). The RT reaction was performed using 200 ng total RNA and dN6 random primers (Inbiohighway). The RNA extraction, RT reaction, and RT-qPCR assays were performed as described previously [45]. The specific oligonucleotide primers listed in Table 1 were used (gene accession numbers are included in the table legend). Reactions were conducted using FastStart Universal SYBR Green Master Mix (Roche Diagnostics GmbH, Mannheim, Germany) and the CFX96 Touch Real-Time PCR Detection System (Biorad, Hercules, CA, USA). Moreover, 18S was chosen as the housekeeping gene. The relative levels of target genes’ mRNA expression were determined for each sample following a mathematical model [46].

### 2.9. Immunocytochemistry Analyses for Characterization of Primary Cell Cultures

The potential contamination of each specific primary cell culture with other testicular cell populations was evaluated by immunocytochemistry. Freshly isolated hamster testicular cell populations (SC, TPC, LC, GC, and tMAC) obtained from immature, young adult, or aged adult Syrian hamsters were fixed in 4% formalin for 10 min at room temperature and were used for the immunodetection of SOX9, AMH, α-SMA, VASA, STAR, and CD68, as previously described [37,38,47]. Cells were washed in PBS followed by a 20 min endogenous peroxidase quenching step (10% methanol, 0.3% H_2_O_2_ in PBS), a permeabilization step (5 min incubation with 0.05% saponin and 5 min 20 µg/mL proteinase K, when needed), and an incubation in blocking solution step (30 min incubation in 5% normal serum in PBS). Incubations with the appropriate antibody/antiserum were carried out overnight in a humidified chamber at 4 °C. The following antibodies were used: monoclonal mouse anti-αSMA (1:100, Sigma-Aldrich #A5228, St. Louis, MO, USA), polyclonal rabbit anti-AMH (1:150; see further details in [48]), polyclonal rabbit anti-SOX9 (1:25, Santa Cruz Biotechnology #sc-20095, Santa Cruz, CA, USA), polyclonal goat anti-VASA (1:100, Santa Cruz Biotechnology #sc-48707), polyclonal rabbit anti-StAR (1:100; see further details in [49]), monoclonal mouse anti-CD68 antibody (1:50, DAKO #M0876, Hamburg, Germany) diluted in incubation buffer (2% horse normal serum for α-SMA and CD68; 2% goat normal serum for AMH, SOX9, and STAR; or 2% donkey normal serum for VASA). Cells were washed with PBS and incubated with biotinylated secondary antiserum: goat anti-rabbit IgG, horse anti-mouse IgG (1:500, Vector Laboratories Inc., Burlingame, CA, USA), or donkey anti-goat IgG (1:500, Jackson Immuno Research Labs Inc., West Grove, PA, USA) for 2 h at room temperature. Immunoreactions were visualized with 0.01% H_2_O_2_ and 0.05% DAB solution (in 0.05 M Tris–HCl) and an avidin–biotin–peroxidase system (Vector Laboratories Inc.). Photographs were taken with a light microscope (Zeiss, Oberkochen, Germany). For control purposes, the first antibody was omitted, and incubation was carried out with 2% normal serum.

### 2.10. Statistical Analysis

Statistical analyses were performed using ANOVA followed by the Student–Newman–Keuls test for multiple comparisons using GraphPad Prism version 6.0 (GraphPad Software, Inc., San Diego, CA, USA). Data are expressed as the mean ± SEM. Differences were considered statistically significant when *p* < 0.05. Different letters denote significant differences between groups (group comparisons are described in each figure/table legend). For TLC studies, spots were quantified by densitometry using the FIJI software v1.53t.(NIH, Bethesda, MA, USA, https://imagej.net/ij/).

## 3. Results

### 3.1. Testes from Immature, Young Adult, or Aged Adult Syrian Hamsters Display Differential Concentrations and Proportions of Polyamines (PAs) and Their Monoacetylated Derivatives (AC-PAs)

**Figure 2:**
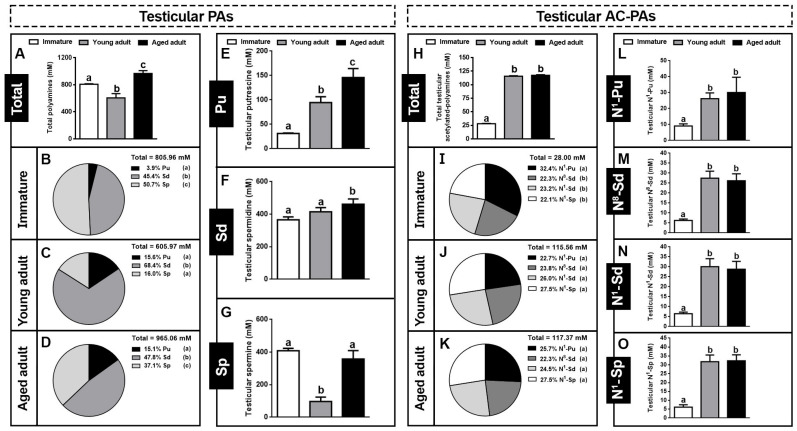
The levels of total PAs (putrescine + spermidine + spermine), individual PAs, total monoacetylated PA derivatives (AC-PAs) (N^1^-putrescine + N^8^-spermidine + N^1^-spermidine + N^1^-spermine), and individual AC-PAs were determined in testes of immature (21 days, *n* = 8), young adult (90 days, *n* = 6), and aged adult (22 months, *n* = 10) Syrian hamsters by thin-layer chromatography (TLC). They are represented as testicular concentrations (mM) for PAs (**A**,**E**–**G**) or AC-PAs (**H**,**L**–**O**) or as a percentage of total PAs (**B**–**D**) or total AC-PAs (**I**–**K**). The graphs represent the mean ± SEM. Different letters denote significant differences (*p* < 0.05; Student–Newman–Keuls test) between age groups (**A**,**E**–**G**,**H**,**L**–**O**) or between types of PAs/AC-PAs within the same age group (**B**–**D**,**I**–**K**).

Testicular levels of total main PAs (putrescine + spermidine + spermine) were significantly different in all three age groups, with the lowest levels recorded in the testes of young animals, followed by the testes of immature animals and, finally, the testes of aged animals (Figure 2A–D). There was a clear age-dependent increase in testicular putrescine levels, with the lowest levels recorded in the testes of immature animals, followed by the testes of young animals and, finally, the testes of aged animals (Figure 2E). Spermidine testicular levels were slightly increased in aged testes compared to immature and young adult testes, although no statistical difference was detected between the last two age groups (Figure 2F). Spermine testicular levels did not differ significantly between the testes of immature animals and aged animals but were highly reduced in young adult testes (Figure 2G). Hence, in both young and aged adult testes, the most prominent PA was spermidine (Figure 2C,D), while spermine was the most abundant in immature testes (Figure 2B).

Testicular levels of total main AC-PAs (N^1^-putrescine + N^8^-spermidine + N^1^-spermidine + N^1^-spermine) were much lower than total main PAs levels in immature, young adult, and aged adult animals (~28.8-fold, ~5.2-fold, and ~8.2-fold, respectively). No differences were detected between young adult and aged adult testes; however, significantly lower levels were observed in the testes of immature animals (Figure 2H–K). All four main AC-PAs were equally represented in the testes of young adult and aged adult hamsters (Figure 2J,K). Only N^1^-Pu levels were slightly higher in immature testes (Figure 2I).

### 3.2. Age-Dependent Expression of Key Genes Involved in Polyamine Metabolism in Syrian Hamster Testes

**Figure 3:**
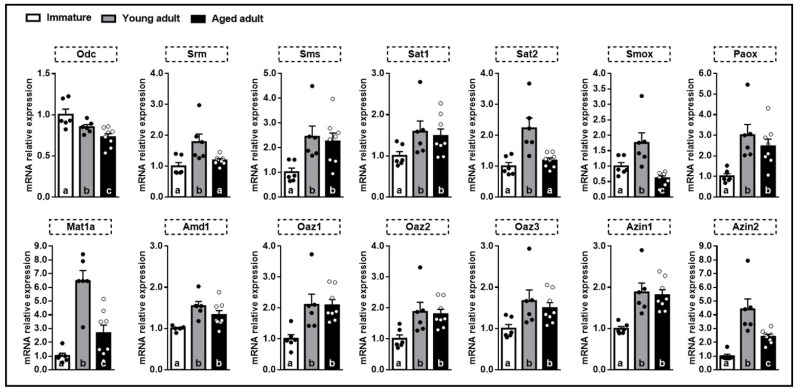
Age-specific relative expression levels of key genes involved in testicular polyamine metabolism. Testicular samples from immature (*n* = 6), young adult (*n* = 6), and aged adult (*n* = 8) Syrian hamsters were evaluated by RT-qPCR. Results for each gene were normalized to 18S and expressed as a fold change relative to the control group (immature), which was assigned a value of 1. The graphs represent the mean ± SEM. Different letters denote significant differences (*p* < 0.05; Student–Newman–Keuls test) between age groups for a specific gene. Odc: ornithine decarboxylase; Srm: spermidine synthase; Sms: spermine synthase; Sat1: spermidine/spermine N^1^-acetyltransferase 1; Sat2: spermidine/spermine N^1^-acetyltransferase 2; Smox: spermine oxidase; Paox: polyamine oxidase; Mat1a: methionine adenosyltransferase 1a; Amd1: S-adenosylmethionine decarboxylase; Oaz1: ornithine decarboxylase antizyme 1; Oaz2: ornithine decarboxylase antizyme 2; Oaz3: ornithine decarboxylase antizyme 3; Azin1: antizyme inhibitor 1; Azin2: antizyme inhibitor 2.

Expression levels of key genes involved in testicular polyamine metabolism differed significantly in all three age groups, mainly for Odc, Smox, Mat1a, and Azin2. Except for Odc, higher expression levels of all genes were detected in young adult testes when compared to immature testes (Figure 3). Lower expression levels of most genes (i.e., Odc, Srm, Sat2, Smox, Mat1a, Azin2) were detected in aged adult testes when compared to young adult testes (Figure 3). The expression levels of some genes (i.e., Sms, Sat1, Paox, Amd1, Oaz1, Oaz2, Oaz3, Azin1) remained unchanged in aged adult testes compared to young adult testes (Figure 3).

### 3.3. Age-Specific Total Intra- and Extracellular PAs Levels in Syrian Hamster Testicular Cell Populations

In order to assess which cell populations of the Syrian hamster testis contributed (and to what extent) to the local production of PAs and AC-PAs, we detected their intracellular and extracellular levels in five representative testicular cell populations. For this purpose, primary cell cultures of Sertoli cells (SC), testicular peritubular cells (TPC), Leydig cells (LC), germ cells (GC), and testicular macrophages (tMAC) were prepared from the testes of immature, young adult, and aged adult hamsters. The potential contamination of each specific primary cell culture with other testicular cell populations was evaluated by immunocytochemistry. In each case, little to no reaction was observed, thus confirming the high purity of each cell population (Figure S1). The intracellular and extracellular levels of PAs and AC-PAs were assessed by TLC under baseline conditions (1 h incubation at 37 °C after purification).

Total intracellular levels of PAs varied significantly with age, mainly in the SC, TPC, and GC populations. In all cell populations, the levels were generally the lowest in immature cells and increased with age (Table 2). However, LC and GC showed lower levels in aged cells than in young adult cells while LC and tMAC showed no significant differences between young adult and aged adult cells (Table 2).

Total extracellular PA levels showed fewer age-related differences within each cell type. Only in LC and tMAC did these values differ significantly at all three ages (Table 2). Levels were highest in aged adult cells in all populations. The lowest levels occurred only in immature LC, GC, and tMAC, whereas SC and TPC showed similarly low levels in immature and young adult cells (Table 2).

Young adult GC showed the highest intracellular/extracellular PA ratio, whereas immature SC showed the lowest ratio (Table 2). Except for tMAC, which showed a clear age-dependent decrease, most cell populations had the lowest ratios in immature cells and higher ratios in young and aged adult cells. SC and TPC showed no detectable differences between young and aged adult cells (Table 2).

### 3.4. Cell-Specific Intra- and Extracellular PAs Levels in Immature Syrian Hamsters

**Figure 4:**
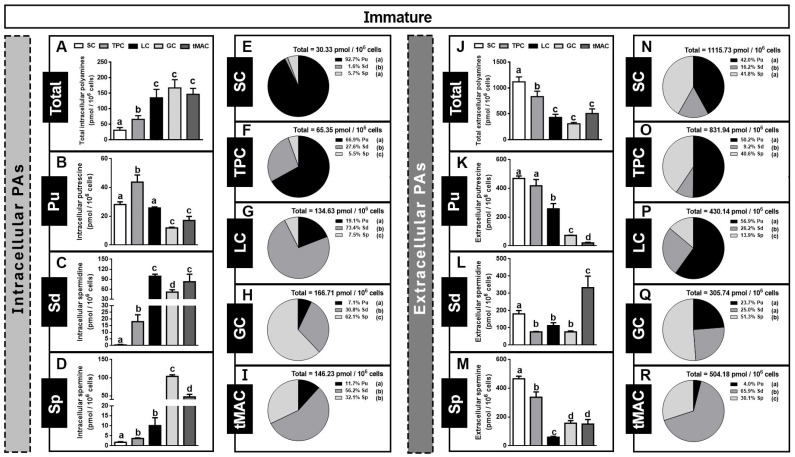
The baseline intracellular (**A**–**I**) and extracellular (**J**–**R**) levels of total (**A**,**J**) PAs (putrescine + spermidine + spermine) or individual (**B**–**D**,**K**–**R**) PAs were determined in primary cell cultures of SC, TPC, LC, GC, and tMAC from immature (*n* = 3) Syrian hamsters by TLC. They are represented as pmol/10^6^ cells (**A**–**D**,**J**–**M**) or as a percentage of total PAs (**E**–**I**,**N**–**R**). The graphs represent the mean ± SEM. Different letters denote significant differences (*p* < 0.05; Student–Newman–Keuls test) between cell types (**A**–**D**,**J**–**M**) or between types of PAs within a specific cell type (**E**–**I**,**N**–**R**).

In immature Syrian hamster testicular cells, total intracellular PAs levels differed significantly among populations and were lowest in SC followed by TPC, LC/GC/tMAC (Figure 4A). Putrescine was lowest in GC/tMAC, followed by SC/LC and TPC (Figure 4B). Spermidine was lowest in SC followed by TPC, GC and LC/tMAC (Figure 4C). Spermine was lowest in SC followed by TPC/LC, tMAC and GC (Figure 4D). The predominant intracellular PAs were putrescine in SC and TPC, spermidine in LC and tMAC, and spermine in GC (Figure 4E–I).

Total extracellular PAs levels also differed significantly and were lowest in LC/GC/tMAC followed by TPC and SC (Figure 4J). Putrescine was lowest in tMAC followed by GC, LC and TPC/SC (Figure 4K). Spermidine was lowest in TPC/LC/GC followed by SC and tMAC (Figure 4L). Spermine was lowest in LC followed by GC/tMAC, TPC and SC (Figure 4M). The predominant extracellular PA species were putrescine and spermine in SC and TPC, putrescine in LC, spermine in GC, and spermidine in tMAC (Figure 4N–R).

### 3.5. Cell-Specific Intra- and Extracellular PAs Levels in Young Adult Syrian Hamsters

**Figure 5:**
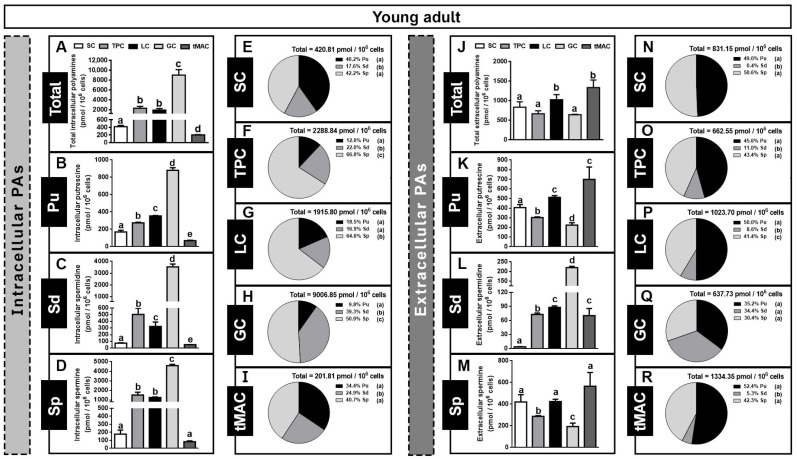
The baseline intracellular (**A**–**I**) and extracellular (**J**–**R**) levels of total (**A**,**J**) PAs (putrescine + spermidine + spermine) or individual (**B**–**D**,**K**–**R**) PAs were determined in primary cell cultures of SC, TPC, LC, GC, and tMACs from young adult (*n* = 3) Syrian hamsters by TLC. They are represented as pmol/10^6^ cells (**A**–**D**,**J**–**M**) or as a percentage of total PAs (**E**–**I**,**N**–**R**). The graphs represent the mean ± SEM. Different letters denote significant differences (*p* < 0.05; Student–Newman–Keuls test) between cell types (**A**–**D**,**J**–**M**) or between types of PA within a specific cell type (**E**–**I**,**N**–**R**).

In young adult Syrian hamster testicular cells, total intracellular PAs levels differed significantly among populations and were lowest in tMAC followed by SC, TPC/LC, and GC (Figure 5A). Putrescine was lowest in tMAC followed by SC, TPC, LC, and GC (Figure 5B). Spermidine was lowest in tMAC followed by SC, LC, TPC, and GC (Figure 5C). Spermine was lowest in tMAC/SC followed by TPC/LC and GC (Figure 5D). Spermine predominated intracellularly in TPC, LC, and GC, whereas putrescine and spermine predominated in SC and tMAC (Figure 5E–I).

Total extracellular PAs levels also differed significantly, and were lowest in SC/TPC/GC followed by LC/tMAC (Figure 5J). Putrescine was lowest in GC followed by TPC, SC and LC/tMAC (Figure 5K). Spermidine was lowest in SC followed by TPC, LC/tMAC and GC (Figure 5L). Spermine showed fewer significant differences, with the highest comparable values in SC/LC/tMAC populations and lower levels in TPC followed by GC (Figure 5M). The predominant extracellular species were putrescine and spermine in SC, TPC, and tMAC and putrescine in LC. All three PA were equally represented in GC, while spermidine was almost undetectable in SC (Figure 5N–R).

### 3.6. Cell-Specific Intra- and Extracellular PAs Levels in Aged Adult Syrian Hamsters

**Figure 6:**
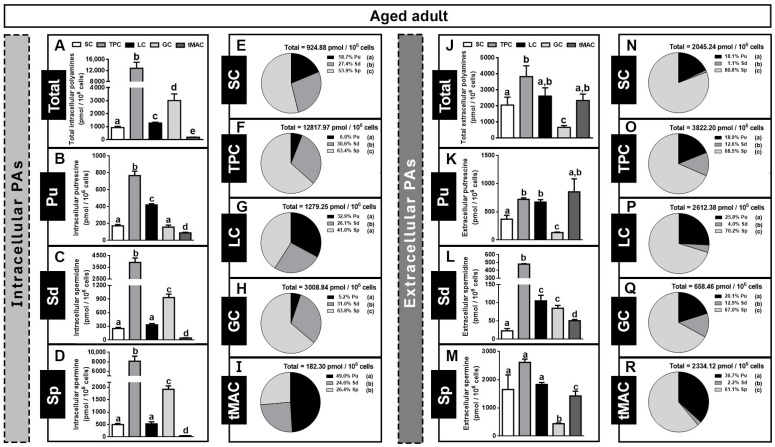
The baseline intracellular (**A**–**I**) and extracellular (**J**–**R**) levels of total (**A**,**J**) PAs (putrescine + spermidine + spermine) or individual (**B**–**D**,**K**–**R**) PAs were determined in primary cell cultures of SC, TPC, LC, GC, and tMAC from aged adult (n = 3) Syrian hamsters by TLC. They are represented as pmol/10^6^ cells (**A**–**D**,**J**–**M**) or as a percentage of total PAs (**E**–**I**,**N**–**R**). The graphs represent the mean ± SEM. Different letters denote significant differences (*p* < 0.05; Student–Newman–Keuls test) between cell types (**A**–**D**,**J**–**M**) or between types of PAs within a specific cell type (**E**–**I**,**N**–**R**).

In aged adult Syrian hamster testicular cells, total intracellular PAs levels varied markedly and were lowest in tMAC followed by SC, LC, GC, and TPC (Figure 6A). Putrescine was lowest in tMAC followed by SC/GC, LC, and TPC (Figure 6B). Spermidine was lowest in tMAC followed by SC/LC, GC, and TPC (Figure 6C). Spermine was lowest in tMAC followed by SC/LC, GC, and TPC (Figure 6D). Intracellularly, putrescine predominated in tMAC, spermine predominated in SC, TPC, and GC, and both putrescine and spermine predominated in LC (Figure 6E–I).

Total extracellular PAs levels were lowest in GC followed by SC/LC/tMAC/TPC (Figure 6J). Putrescine was lowest in GC followed by SC/LC/tMAC/TPC (Figure 6K). Spermidine was lowest in SC followed by tMAC, GC/LC and TPC (Figure 6L). Spermine was lowest in GC followed by tMAC and SC/LC/TPC (Figure 6M). Interestingly, the predominant extracellular species in all cell populations was spermine (Figure 6N–R).

### 3.7. Age-Specific Intra- and Extracellular AC-PAs Levels in Syrian Hamster Testicular Cell Populations

Total intracellular AC-PAs levels differed significantly with age in most populations, mainly in SC, TPC, LC, and GC (Table 3). The levels were highest in young adult cells across all populations. In SC, LC, and GC, aged adult cells showed lower levels than young adult cells (Table 3). In the tMAC, young and aged adult cells showed comparable levels, both higher than in immature cells, where intracellular AC-PAs levels were 1000–10,000-fold lower than in any other cell/age group (Table 3).

Total extracellular AC-PAs levels differed significantly across the three ages in SC, TPC, and LC (Table 3). The levels were lowest in immature cells and highest in aged adult cells across all populations, except in GC and tMAC, where aged and young adult cells showed comparable levels (Table 3).

Immature GC showed the highest intracellular/extracellular AC-PAs ratio, while aged adult LC and SC, and tMAC at all ages, showed the lowest ratios (Table 3). Except for tMAC, whose ratio did not vary with age, all cell populations showed the lowest ratios in aged adult cells and the highest ratios in young adult and immature cells.

### 3.8. Cell-Specific Intra- and Extracellular AC-PAs Levels in Immature Syrian Hamsters

**Figure 7:**
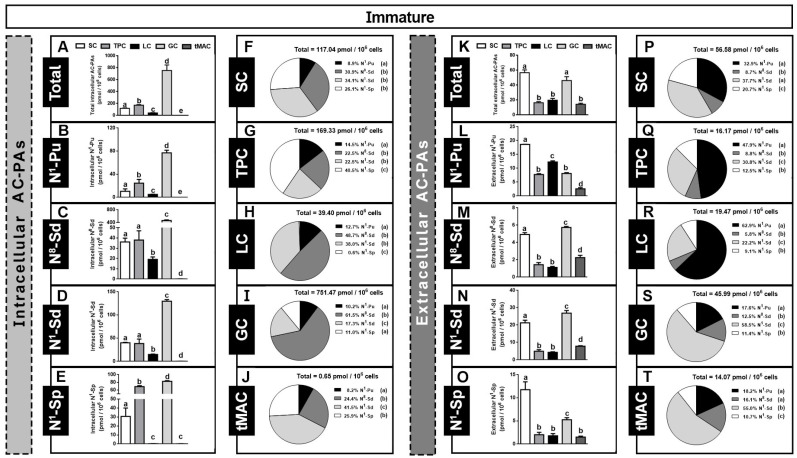
The baseline intracellular (**A**–**J**) and extracellular (**K**–**T**) levels of total (**A**,**K**) AC-PAs (N^1^-Pu + N^8^-Sd + N^1^-Sd + N^1^-Sp) or individual (**B**–**J**,**L**–**T**) AC-PAs were determined in primary cell cultures of SC, TPC, LC, GC, and tMAC from immature (n = 3) Syrian hamsters by TLC. They are represented as pmol/10^6^ cells (**A**–**E**,**K**–**O**) or as a percentage of total PAs (**F**–**J**,**P**–**T**). The graphs represent the mean ± SEM. Different letters denote significant differences (*p* < 0.05; Student–Newman–Keuls test) between cell types (**A**–**E**,**K**–**O**) or between types of AC-PAs within a specific cell type (**F**–**J**,**P**–**T**).

In immature Syrian hamster testicular cells, total intracellular AC-PAs levels differed significantly among populations and were lowest in tMAC followed by LC, SC, TPC, and GC. (Figure 7A). N^1^-Pu was lowest in tMAC followed by LC, SC, TPC, and GC (Figure 7B). N^8^-Sd (Figure 7C) and N^1^-Sd (Figure 7D) showed the same pattern, exhibiting lowest levels in tMAC followed by LC, SC/TPC, and GC. N^1^-Sp was lowest in tMAC/LC followed by SC, TPC, and GC (Figure 7E). The predominant intracellular species were N^1^-Sp in TPC, N^8^-Sd and N^1^-Sd in LC, N^8^-Sd in GC, and N^1^-Sd in tMAC; no AC-PA species predominated in SC, although N^1^-Pu was the least abundant (Figure 7F–J).

Total extracellular AC-PAs levels varied less and were lowest in tMAC/TPC/LC followed by GC/SC (Figure 7K). N^1^-Pu was lowest in tMAC followed by GC/TPC, LC, and SC (Figure 7L). N^8^-Sd (Figure 7M) and N^1^-Sd (Figure 7N), were lowest levels were observed in TPC/LC followed by tMAC, SC and GC. N^1^-Sp was lowest in TPC/LC/tMAC followed by GC and SC (Figure 7O). The predominant extracellular species were N^1^-Pu and N^1^-Sd in SC, N^1^-Pu in TPC and LC, and N^1^-Sd in GC and tMAC (Figure 7P–T).

### 3.9. Cell-Specific Intra- and Extracellular AC-PAs Levels in Young Adult Syrian Hamsters

**Figure 8:**
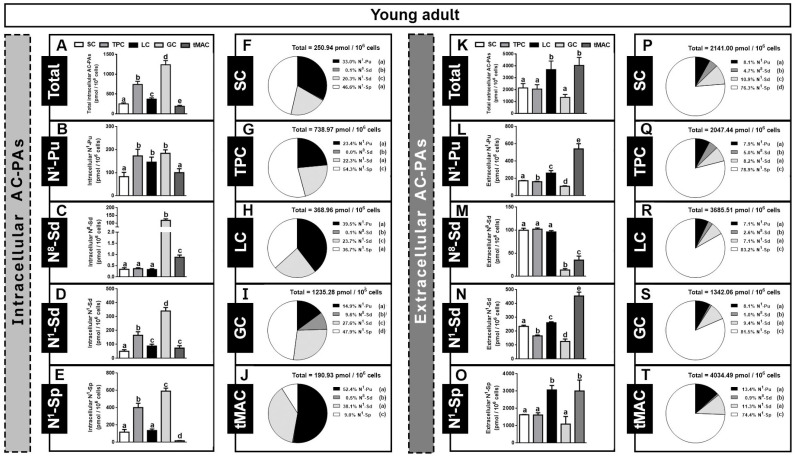
The baseline intracellular (**A**–**J**) and extracellular (**K**–**T**) levels of total (**A**,**K**) AC-PAs (N^1^-Pu + N^8^-Sd + N^1^-Sd + N^1^-Sp) or individual (**B**–**J**,**L**–**T**) AC-PAs were determined in primary cell cultures of SC, TPC, LC, GC, and tMAC from young adult (*n* = 3) Syrian hamsters by TLC. They are represented as pmol/10^6^ cells (**A**–**E**,**K**–**O**) or as a percentage of total PAs (**F**–**J**,**P**–**T**). The graphs represent the mean ± SEM. Different letters denote significant differences (*p* < 0.05; Student–Newman–Keuls test) between cell types (**A**–**E**,**K**–**O**) or between types of AC-PAs within a specific cell type (**F**–**J**,**P**–**T**).

In young adult Syrian hamster testicular cells, total intracellular AC-PAs levels differed significantly among populations and were lowest in tMAC followed by SC, LC, TPC, and GC (Figure 8A). N^1^-Pu was lowest in SC/tMAC followed by TPC/LC/GC (Figure 8B). N^8^-Sd was lowest in SC/TPC/LC followed by tMAC and GC (Figure 8C). N^1^-Sd was lowest in SC followed by LC/tMAC, TPC, and GC (Figure 8D). N^1^-Sp was lowest in tMAC followed by SC/LC, TPC, and GC (Figure 8E). The predominant intracellular species were N^1^-Pu and N^1^-Sp in SC and LC, N^1^-Sp in TPC and GC, N^1^-Pu and N^1^-Sd in tMAC (Figure 8F–J).

Total extracellular AC-PAs levels were lowest in SC/TPC/GC followed by LC/tMAC (Figure 8K). N^1^-Pu was lowest in GC followed by TPC, SC, LC, and tMAC (Figure 8L). N^8^-Sd was lowest in GC followed by tMAC and SC/TPC/LC (Figure 8M). N^1^-Sd was lowest in GC followed by TPC, SC, LC, and tMAC (Figure 8N). N^1^-Sp was lowest in SC/TPC/GC followed by LC/tMAC (Figure 8O). The most abundant extracellular species in all cell populations was N^1^-Sp (Figure 8P–T).

### 3.10. Cell-Specific Intra- and Extracellular AC-PAs Levels in Aged Adult Syrian Hamsters

**Figure 9:**
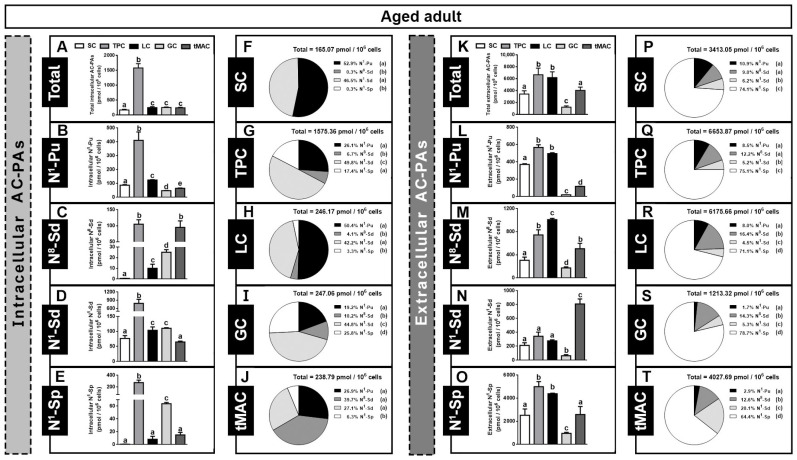
The baseline intracellular (**A**–**J**) and extracellular (**K**–**T**) levels of total (**A**,**K**) AC-PAs (N^1^-Pu + N^8^-Sd + N^1^-Sd + N^1^-Sp) or individual (**B**–**J**,**L**–**T**) AC-PAs were determined in primary cell cultures of SC, TPC, LC, GC, and tMAC from aged adult (*n* = 3) Syrian hamsters by TLC. They are represented as pmol/10^6^ cells (**A**–**E**,**K**–**O**) or as a percentage of total PAs (**F**–**J**,**P**–**T**). The graphs represent the mean ± SEM. Different letters denote significant differences (*p* < 0.05; Student–Newman–Keuls test) between cell types (**A**–**E**,**K**–**O**) or between types of AC-PA within a specific cell type (**F**–**J**,**P**–**T**).

In aged adult Syrian hamster testicular cells, total intracellular AC-PAs levels varied modestly and were lowest in SC, followed by LC/GC/tMAC and TPC (Figure 9A). N^1^-Pu was lowest in GC followed by tMAC, SC, LC, and TPC (Figure 9B). N^8^-Sd was lowest in SC followed by LC, GC, and TPC/tMAC (Figure 9C). N^1^-Sd was lowest in SC/tMAC followed by LC/GC and TPC (Figure 9D). N^1^-Sp was lowest in SC/LC/tMAC followed by GC and TPC (Figure 9E). The predominant intracellular species were N^1^-Pu and N^1^-Sd in SC, N^1^-Sd in TPC and GC, and N^1^-Pu in LC; no AC-PA species predominated in tMAC, although N^1^-Sp was the least abundant (Figure 9F–J).

Total extracellular AC-PAs levels were lowest in GC followed by SC/tMAC and TPC/LC (Figure 9K). N^1^-Pu was lowest in GC followed by tMAC, SC, and TPC/LC (Figure 9L). N^8^-Sd was lowest in GC followed by SC, tMAC/TPC, and LC (Figure 9M). N^1^-Sd was lowest in GC followed by SC/TPC/LC and tMAC (Figure 9N). N^1^-Sp was lowest in GC followed by SC/tMAC and TPC/LC (Figure 9O). Interestingly, the predominant extracellular species in all cell populations was N^1^-Sp (Figure 9P–T).

### 3.11. Age-Specific and Cell-Specific Expression of Key Genes Involved in Polyamine Metabolism in Syrian Hamster Testicular Cell Populations

**Figure 10:**
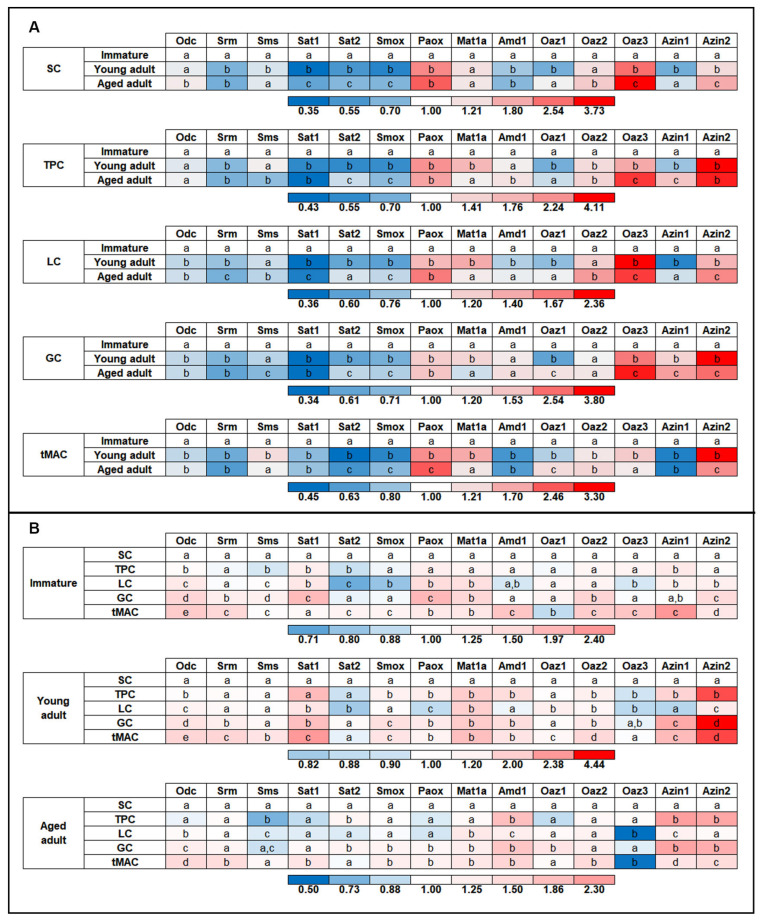
Age-specific and cell-specific relative expression levels of key genes involved in polyamine metabolism were analyzed in primary cell cultures of SC, TPC, LC, GC, and tMAC from immature, (*n* = 4), young adult (*n* = 4), and aged adult (*n* = 4) Syrian hamsters by RT-qPCR. Results for each gene were normalized to 18S and expressed as fold changes relative to the control group: immature (**A**) or SC (**B**), which was assigned a value of 1. Heatmaps represent the mean ± SEM. Different letters denote significant differences (*p* < 0.05; Student–Newman–Keuls test) between age groups within a specific cell type (**A**) or between cell types within a specific age group (**B**).

We analyzed each cell type separately across immature, young adult, and aged adult populations (Figure 10A,B, Figures S2 and S3). We compared age groups within each cell type (Figure 10A and Figure S2) and cell types within each age group, using SC as the reference population (Figure 10B and Figure S3).

#### 3.11.1. Age-Specific Down-Regulation of Odc, Srm, Sat1, Sat2, and Smox mRNA Levels Across All Cell Populations

Odc, Srm, Sat1, Sat2, and Smox showed lower expression in young and aged adult cells than in immature cells across all five cell populations (Figure 10A and Figure S2). In aged adult cells, expression was even lower for Srm in LC and Sat2 in tMAC (Figure 10A and Figure S2).

Sms and Oaz1 were also down-regulated, although their expression varied more across cell types (Figure 10A and Figure S2). Sms expression was lower in aged adult cells than in immature cells. Young adult cells also tended to show lower Sms expression than immature cells, although this difference was statistically significant only in SC and GC. In contrast, young adult tMAC showed higher Sms expression than immature cells (Figure 10A and Figure S2). Oaz1 expression was lower in young adult cells than in immature cells across all five cell populations. In aged adult cells, Oaz1 expression was comparable to the immature levels in SC, TPC, and LC. However, aged adult GC and tMAC showed higher Oaz1 expression than both immature and young adult cells (Figure 10A and Figure S2).

Within each age group, we compared gene expression across cell types, using SC as the reference population (Figure 10B and Figure S3). Sat2 was down-regulated in most young adult cell populations, whereas Sms was down-regulated in most aged adult cell populations (Figure 10B and Figure S3).

#### 3.11.2. Age-Specific Up-Regulation of Paox, Oaz2, Oaz3, and Azin2 mRNA Levels Across All Cell Populations

Paox, Oaz2, Oaz3, and Azin2 showed higher expression in young and aged adult cells than in immature cells across all five cell populations (Figure 10A and Figure S2). Compared with young adult cells, aged adult cells showed even higher expression of Paox in tMAC; Oaz3 in SC, TPC, LC, and GC; and Azin2 in all five cell populations (Figure 10A and Figure S2).

Oaz2 expression was higher in aged adult cells than in immature cells in SC, TPC, LC, and tMAC. In young adult cells, Oaz2 expression was higher than in immature cells only in TPC and tMAC. GC showed no age-dependent regulation of Oaz2 expression (Figure 10A and Figure S2). Mat1a expression was higher in young adult cells than in immature cells, except in SC, where the levels were similar. In aged adult cells, Mat1a expression remained comparable to the immature levels (Figure 10A and Figure S2).

When comparing cell types within each age group (Figure 10B and Figure S3), patterns of up-regulated expression were found in the majority of immature cells (for Paox, Mat1a, and Azin2), in the majority of young adult cells (for Mat1a, Oaz2, and Azin2), and in the majority of aged adult cells (for Mat1a, and Azin2) (Figure 10B and Figure S3).

#### 3.11.3. No Age-Related Regulation of Amd1 and Azin1 mRNA Levels Was Detected in Testicular Cell Populations

In young adult cells, Amd1 and Azin1 expression was lower than in immature cells in SC, LC, TPC, and tMAC, but Amd1 levels in TPC and GC were comparable (Figure 10A and Figure S2). In contrast, young adult GC showed higher Azin1 expression compared to immature GC (Figure 10A and Figure S2).

Aged adult cells showed the greatest variation. Compared to immature cells, Amd1 expression was lower in aged adult SC and tMAC and Azin1 expression was lower in aged adult tMAC (Figure 10A and Figure S2). Azin1 expression was higher in aged adult TPC and GC and Amd1 expression was higher in aged adult tMAC (Figure 10A and Figure S2). Azin1 expression was also significantly higher in aged adult GC than in young adult GC (Figure 10A and Figure S2).

## 4. Discussion

### 4.1. Smox-/Sat1-/Sat2-/Paox-Mediated Polyamine Catabolism May Drive Putrescine Increase and Spermine Decrease During Testicular Maturation

It is envisaged that the lower concentration of total PAs in the testes of young adult animals compared to immature animals is indicative of an increase in PA catabolism. The up-regulation of Smox, Sat1/Sat2, and Paox supports enhanced spermine oxidation, PA acetylation, and back-conversion pathways, providing a plausible explanation for the decrease in spermine and the accumulation of putrescine during testicular maturation. The up-regulation of Paox could also explain the increase in putrescine levels detected in the testes of young adult animals compared to immature animals. Although Odc’s testicular expression was lower in young adult animals than in immature animals, putrescine concentration was higher, indicating that transcriptional regulation alone does not account for putrescine’s abundance. The regulation of ODC expression and function is very complex and requires accessory proteins that have inhibitory (OAZ1/2/3) or activating (AZIN1/2) effects on ODC function. When both antizymes and antizyme inhibitors are present, antizymes have a greater affinity for antizyme inhibitors than for ODC, allowing functional ODC dimers to form [5,50]. Our results showed that Oaz1/2/3 and Azin1/2 testicular expression was higher in young adult animals than in immature animals, suggesting that this regulatory network may help to sustain ODC activity despite reduced Odc transcript levels, thereby contributing to putrescine production. This is consistent with the well-established roles of putrescine in testicular development, spermatogenesis, and sperm motility [51,52]. Despite the higher Srm testicular expression in young adult animals compared to immature animals, spermidine concentration did not increase significantly. A possible explanation might be related to the accumulation of its acetylated forms (N^8^-Sd/N^1^-Sd) due to the increase in Sat1/Sat2 expression. The lower spermine testicular levels were probably not associated with reduced biosynthesis or aminopropyl donor availability, since Sms, Mat1a, and Amd1 expression was induced in young adult testes. Thus, the cooperative action of Smox/Sat1/Sat2/Paox could be key to understanding the variations in tissue PAs/AC-PAs levels during testicular maturation.

### 4.2. Putrescine and Spermidine Increase via Sat1-/Paox-Mediated Polyamine Catabolism Might Be a Feature of Testicular Aging

The tissue levels of PAs in aged adult testes were higher than in immature animals, although the higher levels of spermidine could not be explained by a higher conversion of spermine to spermidine, since the expression of Smox was decreased. However, the up-regulation of Sat1 may have promoted spermine acetylation, therefore increasing N^1^-Sp availability for Paox-mediated conversion to spermidine. This combined action of Sat1/Paox could also account for the higher putrescine levels detected in aged testes, because increased Sat1 expression would favor spermidine acetylation to N^8^-Sd/N^1^-Sd, which can be subsequently converted to putrescine by Paox.

### 4.3. Effects of Cellular Composition of the Testis on Total Polyamine Concentration

The cellular composition of the testis may partly explain the differences in whole-tissue PA abundance. In adult testes, the interstitial compartment represents 10–20% of the tissue, with Leydig cells and tMAC accounting for approximately 1.4% and 0.35%, respectively, whereas the tubular compartment represents 80–90% of the tissue [53]. Within seminiferous tubules, SC + TPC account for 10–15%, while GC represent 85–90% [53]. Thus, age-dependent changes in both cell-specific PA profiles and relative cell abundance likely influence total PA levels. For example, the larger GC fraction in young adult testes may increase their contribution to the total PA content, whereas the higher SC + TPC proportion in immature tubules may give these cells greater weight at earlier ages. Although tMAC numbers increase in aged testes [26], their low relative abundance suggests a limited contribution to total PA levels. In contrast, SC, LC, and GC remain major contributors to aged testicular PA content despite their age-related reported decline [54].

### 4.4. An Age-Dependent Remodeling of Cellular Polyamine Metabolism Promoted Spermine-/N^1^-Spermine-Rich Profiles

The isolated-cell analysis of the intracellular and extracellular PAs content showed that PAs levels were highly cell type-specific and strongly age-dependent. Although no isolated cell population reproduced the overall testicular PA profile, each one contributed differently to the total testicular PAs pool, showing distinct intracellular PA content and extracellular release patterns.

A clear age-related remodeling of intracellular PAs content was detected. In immature cells, the intracellular individual PAs levels were generally lower and more heterogeneous. During maturation, the intracellular individual PAs ranges increased markedly, especially in GC. At this stage, a shift toward spermine predominance in all cell types could be detected, which was maintained during aging. Extracellular PA profiles showed a similar predominance of spermine, whereas extracellular AC-PAs progressively shifted toward N^1^-Sp as the predominant species. In contrast, intracellular AC-PA composition remained more variable, with N^1^-Sd and N^1^-Pu predominating, depending on the cell type.

The relationship between gene expression and cellular PA/AC-PA content is likely determined by the coordinated activity of the entire metabolic pathway, rather than by changes in individual enzymes. Although the specific associations differed among cell populations, the overall expression profiles were broadly consistent with the observed intracellular PA and AC-PA patterns, supporting the contribution of the coordinated regulation of biosynthetic, catabolic, and regulatory enzymes to the age-dependent remodeling of PA metabolism. The higher extracellular N^1^-Sp levels detected in aged cells may reflect higher substrate availability for Sat1/Sat2, mainly in TPC and GC, which also elevated spermine content and increased the expression of these enzymes.

Interestingly, the total intracellular/total extracellular PA ratio differed markedly among cell populations. tMAC were the only cell population in which the ratio decreased in an age-dependent manner, whereas GC displayed the highest intracellular retention of both PAs and AC-PAs. These findings suggest that aging may modify not only intracellular PA metabolism but also cell type-specific PA transport dynamics. Although the molecular mechanisms remain to be established, transporters such as Slc3a2 and Slc22a2, which are critical for PA/AC-PA transport [55,56,57,58], are expressed in Syrian hamster testicular cells (our unpublished data) and represent plausible candidates for regulating these age-dependent changes.

### 4.5. Optimized Protocols Yield Highly Purified, Viable SC and TPC from Adult Testes

In order to assess which cell populations of the Syrian hamster testis contributed (and to what extent) to the local production of PAs and AC-PAs, we performed a more exhaustive analysis for the quantification of intracellular and extracellular levels of PAs and AC-PAs in primary cell cultures of SC, TPC, LC, GC, and tMAC purified from immature, young adult, and aged adult testes. Our group has previously established verified protocols ensuring the preservation of cellular function [27,36,37,38]. Here, we describe a novel protocol for the isolation of SC and TPC from adult (young and aged) animals that yielded highly viable cell populations with minimal GC contamination (Figure S1). Compared with previously reported protocols [59,60,61,62,63,64], our approach relied on longer collagenase digestion, lower collagenase concentration, and an additional discontinuous Percoll density gradient step, allowing the recovery of viable AMH-positive SC and αSMA-positive TPC while minimizing culture-induced alterations in cell function (Figure S1).

### 4.6. Possible Implications of Testicular Putrescine/Spermidine Increase and Cellular Spermine/N^1^-Sp Shift in Testicular Aging and Pathophysiology

Our results establish that, at least at the tissular level, aged testes show higher levels of total PAs than immature and young adult testes. At first glance, these findings could be counterintuitive, because a general decrease in PAs has been reported with age [65,66,67]. However, in many cases, these age-associated declines in PA concentrations described indicate a decline during early life (fetal period or developmental period), and the decrease slows down markedly in adulthood and often does not translate into a significant decrease in healthy adult aged animals or humans [68,69]. In our case, spermine levels remained unchanged, while spermidine and putrescine levels increased significantly, consistent with the enhanced PA catabolism associated with the up-regulation of Sat1/Paox. Increased catabolism may promote reactive oxygen species (ROS) generation through PA oxidation and acetylation [70]. Chronic inflammation can induce the expression of Smox and Sat1/Sat2, thus increasing the probability of generating ROS. According to our new data, Smox was not naturally induced in the testes of aged Syrian hamsters, so the increased catabolism of PAs via Smox might not be a concerning source of ROS under physiological aging. However, increased expression of Sat1/Sat2 and AC-PAs concentrations could trigger the oxidation of AC-PAs (via Paox) while simultaneously generating H_2_O_2_, which could contribute to ROS accumulation and a pro-oxidative status. In addition, increased Sat1/2 activity has been associated with metabolic cycles that consume ATP and acetyl-CoA [71]; therefore, induced PA acetylation might have a great impact on cellular energy, lipid, and carbohydrate metabolism as well.

Our group has previously reported that aged (22-month-old) Syrian hamster testes exhibited a low-grade inflammatory status together with a pro-oxidative status, decreased autophagy, and reduced DNA repair capability [26,27,28]. Moreover, we detected similar alterations in human testicular biopsies from men suffering from idiopathic infertility (primarily Sertoli cell-only syndrome and hypospermatogenesis) [29,30,31,32,33,34,35]. Therefore, our preliminary findings in aged Syrian hamsters (as well as potential new data generated in the future) may represent an additional mechanism contributing to these age- and pathological-associated changes, supporting the relevance of the aged Syrian hamster as a model for studying male idiopathic infertility.

Despite these potentially deleterious effects of enhanced PA catabolism, PAs also exhibit well-established anti-inflammatory and anti-oxidant properties that may counteract age-related cellular damage [72,73,74]. Spermidine and spermine efficiently scavenge ROS due to their positive charges, which allow them to interact with alkyl groups, and hydroxyl, peroxyl, and superoxide radicals [75]. In vitro and in vivo studies have shown that PAs depletion induces ROS accumulation and cell damage, while exogenous spermidine can activate autophagy and contribute to redox homeostasis [76,77]. Accordingly, accumulating evidence supports a role for PAs in autophagy, which is a central cellular quality-control mechanism [5]. However, increased PAs catabolism, regardless of whether it happens in the context of elevated PAs levels, has also been associated with enhanced apoptosis [5]. Hence, the contribution of PA to cell physiology or pathophysiology could depend on the balance between their cytoprotective actions and the potentially deleterious consequences of their catabolism. As part of their anti-inflammatory actions, PAs have been shown to promote an anti-inflammatory M2 phenotype in macrophages and to inhibit pro-inflammatory cytokine production (e.g., IL1β, IL6, TNFα) while inducing anti-inflammatory cytokine secretion (e.g., IL10), mostly in immune cell types (e.g., microglia and monocytes) [78,79,80,81,82]. Thus, we cannot rule out the possibility that the increase in PAs concentrations and production capacity in aged testes could also be an extra compensatory mechanism to provide additional ROS scavenger molecules and the activation of anti-inflammatory, anti-oxidative, and pro-autophagic pathways, aiming to counteract the ongoing significant alterations and preserve testicular homeostasis during aging.

### 4.7. Future Directions

Mapping out which PAs are predominant/lacking in each cell type (and developmental age) will allow us to perform further studies on the roles of such species in general cellular responses (e.g., apoptosis, autophagy, etc.) or more specific cellular responses more accurately (e.g., steroidogenesis in Leydig cells, lactate production in Sertoli cells, contractility in testicular peritubular cells, maturation/spermatogenesis progression in germ cells, M1/M2 profile [pro-/anti-inflammatory] or phagocytic activity in testicular macrophages). Bearing in mind that there is clear parallelism between the changes taking place in the aged testis and the alterations that occur in the testes of infertile men, the data that could potentially be generated from aged Syrian hamsters (in which all of these cellular processes are deregulated) could also help to advance studies on male idiopathic infertility and may reveal new possible targets with therapeutic potential to slow testicular aging or alleviate male idiopathic infertility.

## 5. Conclusions

Taken together, the most relevant conclusions of our work are as follows: (1) at a tissular level, there is an age-associated increase in PA concentration with a spermidine predominance; (2) different cell populations contribute unequally to PAs production and release, and these contributions change substantially with age; (3) the age-related remodeling of intracellular and extracellular PAs content shows more dissimilar abundance patterns in immature animals and less dissimilar abundance patterns in adult aged animals; (4) at a cellular level (intra- and extracellularly), aging is associated with a shift toward spermine- and N^1^-Sp-rich profiles; (5) the increased catabolism of PAs might be an age-related hallmark in the testis; (6) targeting PA catabolic enzymes might have novel therapeutic potential to alleviate aging-associated (or idiopathic infertility-associated) testicular alterations.

## Figures and Tables

**Table 1:** Oligonucleotide primers used for RT-qPCR analyses.

Gene	Primer Sequence 5′–3′	Amplicon Size
Odc	Fw: AAGTATGCCGCCAGTAACGG Rv: GGCAATCCGCAAAACCAACT	109 bp
Srm	Fw: AAGCACCCCTCTGTGGAG Rv: CATGAACTCAAAGCCATC	144 bp
Sms	Fw: TTTCAGGAGCAGGGGATG Rv: CAGCAACACCAATCCATG	126 bp
Sat1	Fw: AGGTTGCCATGAAGTGTC Rv: CTTGAAGAGCCTCCATCC	131 bp
Sat2	Fw: CGAGAAACTGTCCCATCA Rv: GATGAAGTAGTACAGCCC	160 bp
Smox	Fw: GTACCTGAAGGTGGAGAGC Rv: CCCGAGGGGATGATGTGA	114 bp
Paox	Fw: GAGCCACCACTGCCTGCC Rv: TTCCCACACCACCTGGAT	132 bp
Mat1a	Fw: GGAGAGGGGCATCCAGAT Rv: CCATACCTGTCTTGCACA	118 bp
Amd1	Fw: AGTGGAATTCGTGACCTG Rv: TTCTGGTTCTGGAGTGATGTG	129 bp
Azin1	Fw: GAAGTGCTGTATAGAGAG Rv: GCCACCAAGCCTATGTCT	134 bp
Azin2	Fw: AGCATGGCTGGCTATCTG Rv: GCAGAAGTGCTTCCTCAC	133 bp
Oaz1	Fw: CGGATGGTGAAATCCTCC Rv: GCTGTGCTGACTAAGAAG	126 bp
Oaz2	Fw: GCCTGACAGGGACTTAGC Rv: CCTCGGTGGCTGTGCTCA	124 bp
Oaz3	Fw: ACCAATGTGGACTCTGTG Rv: TGAAGATGACATTGTCCC	142 bp
18S	Fw: ACACGGACAGGATTGACAGATT Rv: CGTTCGTTATCGGAATTAACCA	110 bp

Odc: ornithine decarboxylase (XM_040730739.1); Srm: spermidine synthase (XM_005079325.4); Sms: spermine synthase (XM_005139217.4); Sat1: spermidine/spermine N^1^-acetyltransferase 1 (XM_040743747); Sat2: spermidine/spermine N^1^-acetyltransferase 2 (XM_013111439.3); Smox: spermine oxidase (XM_040746331.1); Paox: polyamine oxidase (XM_040752037.1); Mat1a: methionine adenosyltransferase 1a (XM_013121174.3); Amd1: S-adenosylmethionine decarboxylase (NM_001281650.1); Azin1: antizyme inhibitor 1 (XM_040730313.1); Azin2: antizyme inhibitor 2 (XM_040756587.1); Oaz1: ornithine decarboxylase antizyme 1 (NM_001281321.2); Oaz2: ornithine decarboxylase antizyme 2 (NM_001290055.1); Oaz3: ornithine decarboxylase antizyme 3 (NM_001290483.1); 18S: 18S ribosomal RNA (NR_003278.3).

**Table 2:** Intracellular and extracellular levels of total PAs (putrescine + spermidine + spermine) were determined in primary cell cultures of SC, TPC, LC, GC, and tMAC from immature (*n* = 3), young adult (*n* = 3), and aged adult (*n* = 3) Syrian hamsters by TLC. Ratios (intracellular to extracellular) were calculated from total level data.

Cell Population	Age	Total Intracellular PAs (pmol/10^6^ Cells)	Total Extracellular PAs (pmol/10^6^ Cells)	Ratio (In/Out)
SC	Immature	30.33 ± 9.01 ^a^	1115.73 ± 95.73 ^a^	0.03 ± 0.01 ^a^
	Young adult	420.81 ± 33.06 ^b^	831.15 ± 136.87 ^a^	0.53 ± 0.32 ^b^
	Aged adult	924.88 ± 97.64 ^c^	2045.24 ± 495.89 ^b^	0.55 ± 0.42 ^b^
TPC	Immature	65.35 ± 11.74 ^a^	831.94 ± 103.11 ^a^	0.08 ± 0.07 ^a^
	Young adult	2288.84 ± 379.85 ^b^	662.55 ± 74.17 ^a^	3.44 ± 0.47 ^b^
	Aged adult	12,817.97 ± 2133.11 ^c^	3822.20 ± 676.49 ^b^	3.37 ± 0.42 ^b^
LC	Immature	134.63 ± 27.34 ^a^	430.14 ± 59.14 ^a^	0.32 ± 0.09 ^a^
	Young adult	1915.80 ± 300.34 ^b^	1023.70 ± 129.22 ^b^	1.88 ± 0.22 ^b^
	Aged adult	1279.25 ± 54.82 ^b^	2612.38 ± 508.74 ^c^	0.49 ± 0.14 ^c^
GC	Immature	166.71 ± 25.52 ^a^	305.74 ± 27.40 ^a^	0.56 ± 0.18 ^a^
	Young adult	9006.85 ± 1102.98 ^b^	637.73 ± 9.46 ^b^	14.52 ± 0.93 ^b^
	Aged adult	3008.84 ± 509.62 ^c^	658.46 ± 111.73 ^b^	4.63 ± 0.45 ^c^
tMAC	Immature	146.23 ± 18.80 ^a^	504.18 ± 90.48 ^a^	0.29 ± 0.04 ^a^
	Young adult	201.82 ± 9.28 ^b^	1334.35 ± 191.24 ^b^	0.16 ± 0.12 ^b^
	Aged adult	182.30 ± 14.38 ^b^	2334.12 ± 399.14 ^c^	0.08 ± 0.05 ^c^

Mean ± SEM. Different letters denote significant differences (*p* < 0.05; Student–Newman–Keuls test) between age groups for each specific cell type.

**Table 3:** Intracellular and extracellular levels of total AC-PAs (N^1^-Pu + N^8^-Sd + N^1^-Sd + N^1^-Sp) were determined in primary cell cultures of SC, TPC, LC, GC, and tMAC from immature (*n* = 3), young adult (*n* = 3), and aged adult (*n* = 3) Syrian hamsters by TLC. Ratios (intracellular to extracellular) were calculated from total levels data.

Cell Population	Age	Total Intracellular AC-PAs (pmol/10^6^ Cells)	Total Extracellular AC-PAs (pmol/10^6^ Cells)	Ratio (In/Out)
SC	Immature	117.04 ± 6.57 ^a^	56.58 ± 3.67 ^a^	2.09 ± 0.23 ^a^
	Young adult	250.94 ± 24.76 ^b^	2141.00 ± 366.82 ^b^	0.12 ± 0.14 ^b^
	Aged adult	165.07 ± 23.61 ^c^	3413.05 ± 558.09 ^c^	0.05 ± 0.03 ^c^
TPC	Immature	169.33 ± 9.32 ^a^	16.17 ± 1.46 ^a^	10.72 ± 1.02 ^a^
	Young adult	738.97 ± 82.24 ^b^	2047.44 ± 368.43 ^b^	0.36 ± 0.14 ^b^
	Aged adult	1575.36 ± 144.96 ^c^	6653.87 ± 1115.17 ^c^	0.24 ± 0.13 ^c^
LC	Immature	39.40 ± 4.37 ^a^	19.47 ± 2.56 ^a^	2.02 ± 0.16 ^a^
	Young adult	368.96 ± 33.18 ^b^	3685.51 ± 716.37 ^b^	0.10 ± 0.09 ^b^
	Aged adult	246.17 ± 30.54 ^c^	6175.66 ± 961.69 ^c^	0.04 ± 0.03 ^c^
GC	Immature	751.47 ± 92.22 ^a^	45.99 ± 5.17 ^a^	16.42± 0.43 ^a^
	Young adult	1235.28 ± 105.31 ^b^	1342.06 ± 253.64 ^b^	1.40 ± 1.04 ^b^
	Aged adult	247.06 ± 18.11 ^c^	1213.32 ± 219.82 ^b^	0.21 ± 0.06 ^b^
tMAC	Immature	0.65 ± 0.04 ^a^	14.07 ± 1.43 ^a^	0.05 ± 0.04 ^a^
	Young adult	190.93 ± 23.26 ^b^	4034.49 ± 674.42 ^b^	0.05 ± 0.07 ^a^
	Aged adult	238.79 ± 16.54 ^b^	4027.69 ± 547.73 ^b^	0.07 ± 0.15 ^a^

Mean + SEM. Different letters denote significant differences (*p* < 0.05; Student–Newman–Keuls test) between age groups for each specific cell type.

## Data Availability

The original contributions presented in this study are included in the article/Supplementary Material. Further inquiries can be directed to the corresponding author.

## Supplementary Materials

The following supporting information can be downloaded at https://www.mdpi.com/article/10.3390/biology15141184/s1. Figure S1: Characterization of primary cell cultures of testicular cells from immature, young adult, and aged adult Syrian hamsters; Figure S2: Age-dependent relative expression levels of key genes involved in polyamine metabolism in primary cell cultures of testicular cells. Figure S3: Cell-specific relative expression levels of key genes involved in polyamine metabolism in primary cell cultures of testicular cells.

## Abbreviations

The following abbreviations are used in this manuscript:

AC-PAs	Polyamine monoacetylated derivatives
Azin1	Antizyme inhibitor 1
Azin2	Antizyme inhibitor 2
Amd1	S-adenosylmethionine decarboxylase
FBS	Fetal bovine serum
HBSS	Hank’s Balanced Salt Solution
GC	Germ cell
LC	Leydig cell
mM	Millimolar
Mat1a	Methionine adenosyltransferase 1a
N^1^-Pu	N^1^-acetylputrescine
N^8^-Sd	N^8^-acetylspermidine
N^1^-Sd	N^1^-acetylspermidine
N^1^-Sp	N^1^-acetylspermine
Oaz1	Ornithine decarboxylase antizyme 1
Oaz2	Ornithine decarboxylase antizyme 2
Oaz3	Ornithine decarboxylase antizyme 3
Odc	Ornithine decarboxylase
PAs	Polyamines
Paox	Polyamine oxidase
RT-qPCR	Real-time quantitative reverse transcription polymerase chain reaction
ROS	Reactive oxygen species
SAM	S-adenosylmethionine
Sat1	Spermidine/spermine N^1^-acetyltransferase 1
Sat2	Spermidine/spermine N^1^-acetyltransferase 2
SC	Sertoli cell
Smox	Spermine oxidase
Sms	Spermine synthase
Srm	Spermidine synthase
TLC	Thin-layer chromatography
tMAC	Testicular macrophage
TPC	Testicular peritubular cell
