# Supplementary figures and images for: Age- and Cell-Specific Regulation of Testicular Polyamine Metabolism Promotes Increased Catabolism During Maturation and Aging in Syrian Hamsters
Source: Biology (Basel). 2026 Jul 17;15(14):1184. doi: 10.3390/biology15141184 (PMC13403433; doi:10.3390/biology15141184)

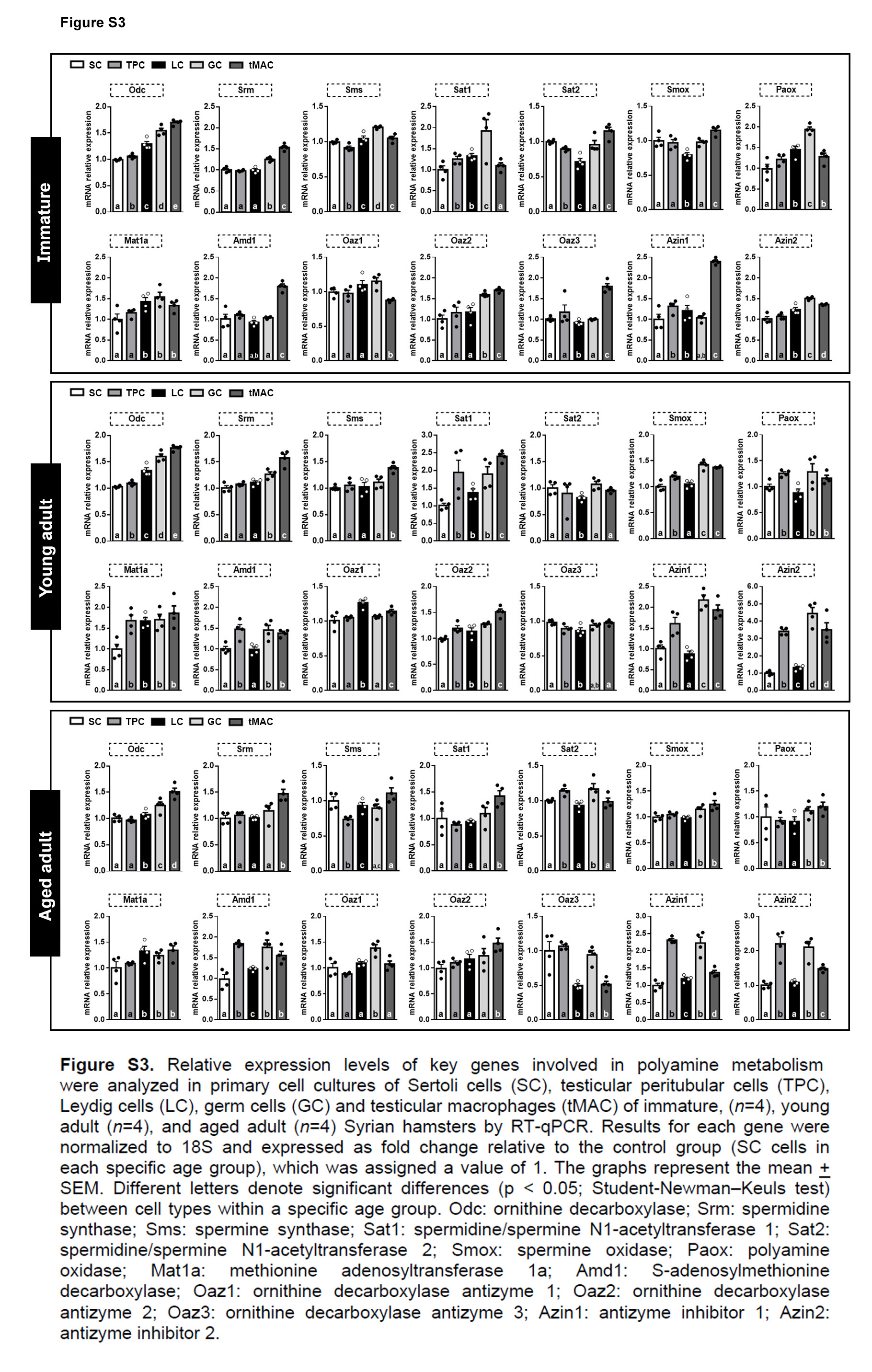

Supplement: Supplementary file 1 [file biology-15-01184-s001.zip › Fig. S3.jpg]

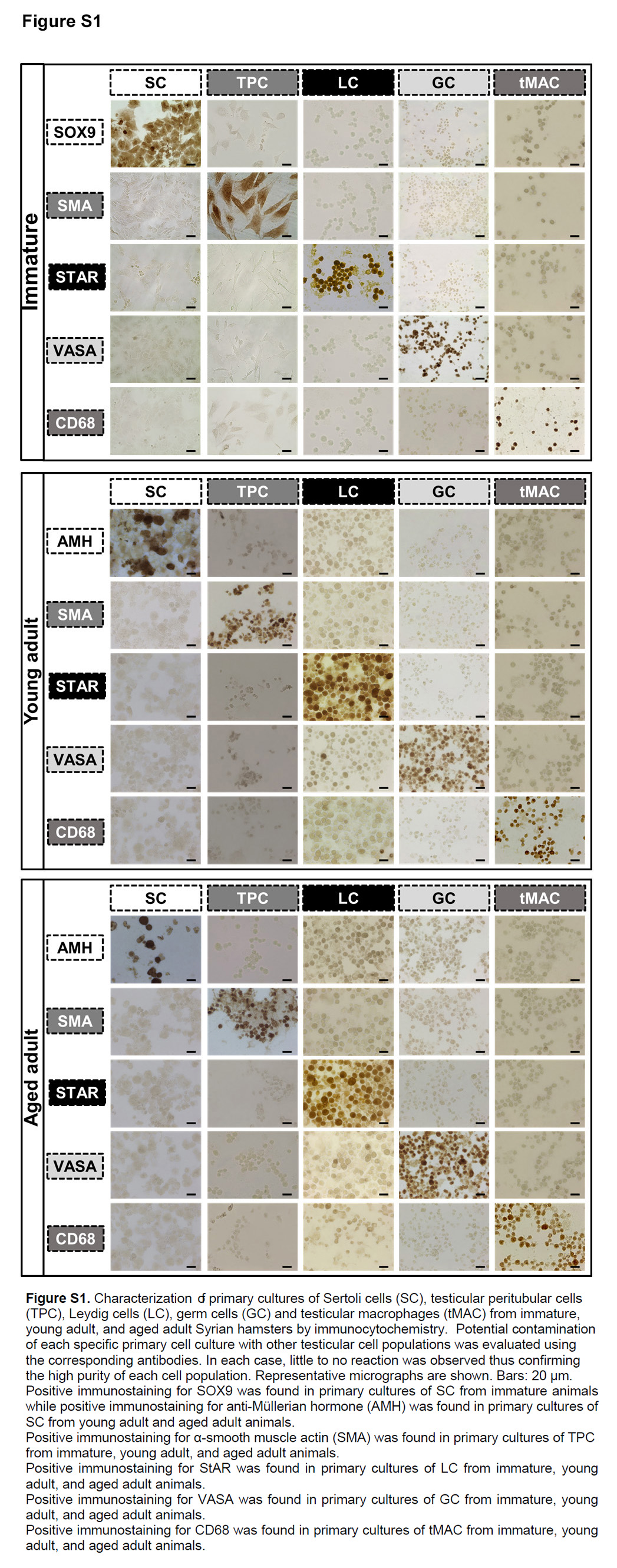

Supplement: Supplementary file 1 [file biology-15-01184-s001.zip › Fig. S1.jpg]

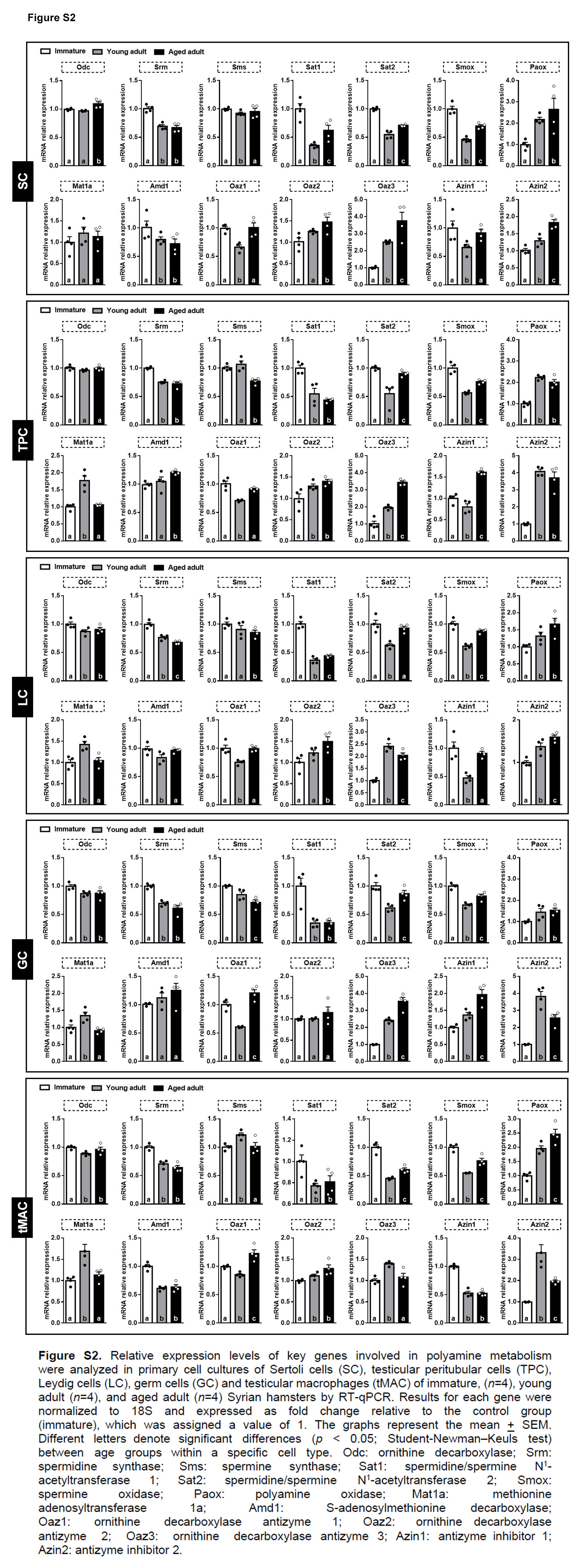

Supplement: Supplementary file 1 [file biology-15-01184-s001.zip › Fig. S2.jpg]
